# Cloning-free regulated monitoring of reporter and gene expression

**DOI:** 10.1186/1471-2199-10-20

**Published:** 2009-03-08

**Authors:** Latifa al-Haj, Wijdan Al-Ahmadi, Maher Al-Saif, Omer Demirkaya, Khalid SA Khabar

**Affiliations:** 1 Program in Biomolecular Research, King Faisal Specialist Hospital and Research Center, Riyadh 11211, Saudi Arabia; 2 Department of Biomedical Physics, King Faisal Specialist Hospital and Research Center, Riyadh 11211, Saudi Arabia

## Abstract

**Background:**

The majority of the promoters, their regulatory elements, and their variations in the human genome remain unknown. Reporter gene technology for transcriptional activity is a widely used tool for the study of promoter structure, gene regulation, and signaling pathways. Construction of transcriptional reporter vectors, including use of cis-acting sequences, requires cloning and time-demanding manipulations, particularly with introduced mutations.

**Results:**

In this report, we describe a cloning-free strategy to generate transcriptionally-controllable linear reporter constructs. This approach was applied in common transcriptional models of inflammatory response and the interferon system. In addition, it was used to delineate minimal transcriptional activity of selected ribosomal protein promoters. The approach was tested for conversion of genes into TetO-inducible/repressible expression cassettes.

**Conclusion:**

The simple introduction and tuning of any transcriptional control in the linear DNA product renders promoter activation and regulated gene studies simple and versatile.

## Background

Transcription occurs due to many different signaling pathways leading to activation of transacting factors that regulate promoter activity. Transcriptional deregulation has been implicated in several diseases such as cancer and inflammatory conditions, and many transcriptional factors themselves are drug targets. For example, the NF-κB signaling pathway has been increasingly seen as a promising target for pharmacological intervention, especially in inflammation and cancer, where the pathway is often constitutively active [1]. The Reporter assays offer an important tool in assessing transcription and gene expression changes due to many extra- and intracellular events including modulation by receptor agonists, intracellular signaling events, and inhibitor compounds. In transcriptional reporter systems, cloning of promoters and inclusion of transcriptional control elements such as transcriptional factor sites and their variations, e.g., mutations and insertions/deletions are time-demanding efforts. Likewise, regulated transcriptional systems, such as the tetracycline-resistance operon (TetO)-based system, requires cloning of the TetO upstream or downstream of the TATA box of a reporter or other gene product [2]. In this report, we describe a simple cloning-free system to construct reporter linear DNA by PCR that is transcriptionally controllable and useful in transcription and regulated gene studies.

A large number of transcriptional control elements, estimated to be in the vicinity of 3000, their genetic variations, and regulatory clusters (>100,000) [3-5] were recently found due to large scale sequencing, functional genomics, and computational prediction studies. Thus, a need for a simplified approach as opposed to cloning and plasmid-based techniques would be desirable. Although high throughput applications of promoter studies have been recently developed [6,7], there is a constant requirement for studying minimal transcriptional regions, to introduce genetic variations, and to manipulate position and arrangements of transcriptional cis-acting elements of single or multiple genes. Here, we utilized mammalian transfection of transcriptionally-regulatable reporter PCR products that are generated with any variation of the transcriptional control elements, i.e., by a simple and cloning-free step. The utility of this strategy is flexible and can be used in constructing as many as desired promoter and transcriptional element manipulations with a larger throughput than conventional cloning. Additionally, the conversion of any gene in an expression vector to Tet-inducible/repressible system without any cloning indicates the notable simplicity of the described approach.

## Results

### Transcriptionally-Controllable Cloning-Free Reporter Assay

**Figure 1:**
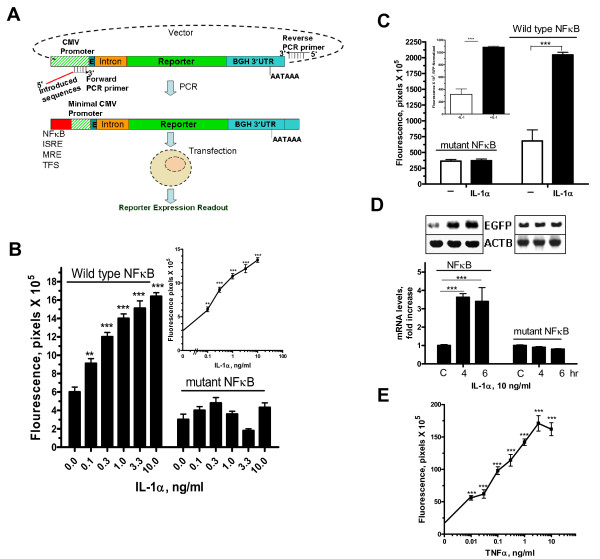
**Schematic representation of the cloning-free reporter construction and assay and use in inflammation model. (A) **An expression plasmid harboring a reporter cDNA is used as a template for PCR. Forward primer sequence contains 3' end that targets a minimal promoter region and 5'end that accommodates cis-acting sites. The reverse universal primer targets a region downstream of the polyA site. Purified PCR products are transfected into mammalian cells to express the reporter.**(B) **HEK293 cells in 96-well plates were transfected with 75 ng of purified PCR products in which the forward primer contains two copies of NF-κB site or a mutant form (**Table 1**, *SEQ 1 and 2*). After 20 hr, the cells were treated with IL-1α (log_0.5_) for additional 20 hrs. **(B, insert)**. Dose-response relationships between IL-1 and NF-κB mediated GFP reporter activity (normalized to basal levels of mutant NF-κB reporter levels). **(C) **EGFP activity was measured after 4 hr of IL-1 treatment with or without normalization (**insert**) with non-responsive RFP-PCR product. All data above are Mean ± SEM (quadruplicate) of one of three experiments. **p < 0.01, *** < 0.001. **(D) **Total RNA was extracted from HEK293 cells that were transfected with NF-κB responsive or mutant NF-κB-bearing PCR products in the absence or presence of IL-α (10 ng/ml) for 4 or 6 hrs. RT-PCR was performed using primers specific to EGFP as described in Methods. PCR products were run on gel, and β-actin normalized signal intensities of ethidium bromide-stained products were quantitated using AlphaEase software. Data are from three experiments.**(E**) Dose-dependent curve of TNF-α action on reporter activity produced from NF-κB responsive linear PCR construct.

The reporter assay is dependent on amplification of a functional reporter cassette from a vector with efficient mammalian expression modules, e.g., strong promoter, intron, and the bovine growth hormone (bgh) 3'UTR (Figure 1A). When transfected as a linear product, it is sufficient to express the reporter. The design of the PCR forward primer, through a 3' end sequence, allows flexibility in targeting any position in the source vector, e.g., minimal promoter, while the 5' end part includes other desired transcriptional control sequences, e.g., transcriptional factor site or its variants (Figure 1A). We have used this approach with a number of different strategies. Each is described here with a demonstration of its utility in several applications of transcriptional studies, particularly in inflammation and innate immunity models.

### Inflammatory response of NF-κB-regulated PCR reporter product

To demonstrate the applicability of the described approach in a commonly-studied inflammation response model, we constructed, by a single PCR, a linear construct using a forward primer that includes two copies of the consensus NF-κB site and 3' end sequence that targets a region in the CMV promoter in vector (Table 1). As a control, mutant NFκB sites were also incorporated in a different linear construct. Thus, a PCR product was produced containing the NFκB-responsive minimal promoter upstream of the EGFP reporter along with a 3' UTR. After transfection onto HEK293 cells overnight, IL-1α was added. IL-1α increased, in a dose-dependent manner, the EGFP reporter activity produced from the PCR product that harbors the NF-κB-minimal promoter (Figure 1B). In contrast, IL-1α did not upregulate any reporter activity from the linear PCR construct made with the mutant NFκB site (Figure 1B). There appears to be a higher (~2-fold) constitutive activity of NF-κB when compared to mutant NF-κB PCR products (*ANOVA*, p < 0.01). A five-fold increase (Figure 1B, insert) in the transcriptional induction was seen with the highest dose of IL-1α (10 ng/ml), when readings were normalized to background basal levels. There was also appreciable promoter activity (~3–4-fold) due to NF-κB activation as assessed with fluorescence signals after only four hours of treatment (Figure 1C) indicating the sensitivity of the transcriptionally-modulated linear reporter. Normalization by co-transfection with red fluorescent protein (RFP) yielded the same results (Figure 1C, insert). The transcriptional induction was also seen at mRNA level. IL-1α induced significant EGFP mRNA expression (4-fold) when assayed at 4 and 6 hr post-treatment (Figure 1D).

**Table 1:** Sequences in forward primers used for generation of the transcriptionally-regulatable PCR products

**SEQ**	**Forward Primer**	**PCR product will contain**	**I-TFS**
1	5' AG*GGGACTTTCC*TGAGTCAA*TAGGGACTTTCC*AATGCCAAAATGTCGTAACAACTC3'	NF-κB responsive Minimal Promoter	NF-κB
2	5' AGGCGACTCTCCTGAGTCAATACTCACTCT CCAATGCCAAAATGTCGTAACAACTC3'	Mutant NF-κB/Minimal Promoter	NF-κB mutant
3	5'CAATAGGGACTTTCCCATGGGACTTTCCCAGGGGACTTTCCTGAGTCAATAGGGACTTTCCAATGGGTAGGCGTGTACGGTG3'	NF-κB (4×) responsive Minimal Promoter (-53)	NF-κB 4×
4	5' CTGCGCTCAGGGGACCTTGCGCCCGGCCCTTCTGCTGCACACAGCCCACCCCAAAATGTCGTAACAACTC3'	Metal response Minimal Promoter	MT1F-MRE
5	5'ACTCAGCGGGCTGGGTGCAAGGGCGGGGCGGGGCGTCTGCGCCCGGCCCCGCCAAAATGTCGTAACAACTC 3'	Metal response Minimal Promoter	MT1G-MRE
6	5' AG*CTTTAGTTTCAC*TTTC*CCCTTTCGGTTTC*CCTAGGTTTCCAACCAATGCCAAAATGTCGTAACAACTC3'	IFN-responsive Promoter	ISRE
7	5' CTTGGGGCGGGGCTGCGGAGATCTCGCGGCGCTTGGCGTGCTATAAAAGCAGAGCTCGTTTAGTGA3'	RPS2 Minimal Promoter	-
8	5' GAAGGCACTAGAAGAGGCGGTCGGTGCATAGCGTCACCTCCTAAACTCGCAGAGCTCGTTTAGTGA3'	RPL39 Minimal Promoter	-
9	5'GTAAATGACATAGGAAAACTGAAAGGGAGAAGTGAAAGTGGGAAATTCCTCTGAATAGAGAGAGGACCATCTCATATAAATAGCAGAGCTCGTTTAGTGA3'	Minimal virus-induced IFNB prom.	VRE
10	5'ACCAGGTCCCTATCAGTGATAGAGATCCTCCCTTCAGTGATAGAGACCAAAATGTCGTAACAACTC3'	TetO_2_-Minimal Promoter (-95)	TetO
11	5'ACCAGGTCCCTATCAGTGATAGAGATCCTCCCTATCAGTGATAGAGAGGTAGGCGTGTACGGTG3'	TetO_2_-Minimal Promoter (-53)	TetO
12	5'CATCCCTATCAGTGATAGACCATCCCTATCAGTGATAGACCATCCCTATCAGTGATAGACGGTAGGC GTGTACGGTG3'	TetO_3_-Minimal Promoter (-53)	TetO

The reverse universal primer targets a region downstream of the polyA site.

I-TFS: inducible transcriptional factor sites (italics).

Underlined is the 3' end region of the primer complementary to the vector template.

The minimal promoter of SEQ 1–6 and 10–12 is derived from CMV minimal promoter (Figure 3B).

There was a dose-dependent increase in EGFP fluorescence from the transfected linear PCR construct that harbors the two NFκB sites in response to another pro-inflammatory cytokine, TNF-α (Figure 1E). A maximal response of 6-fold increase was seen with 3 ng/ml of TNF-α. As with IL-1α, the transfected linear PCR products that incorporate mutant NFκB were not responsive to TNF-α (data not shown).

### Dynamic response of promoter activity from PCR reporter products

**Figure 2:**
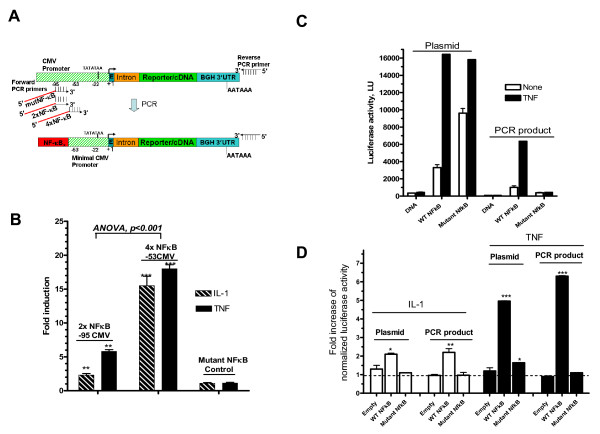
**Dynamic response of the activity of reporter expressed from PCR product. (A) **The reporter expression plasmid was used as a template for PCR in which the forward primers target either -53 or -95 sequences of the CMV promoter and includes two or four copies of NF-κB or a mutant NF-κB control (**Table 1**, *SEQ. 1–3*). **(B) **Fluorescence levels from EGFP PCR products that contain two, four, or mutant copies of NF-κB were quantitated as described above.**(C) **HEK293 cells were transfected with either NfκB-firefly luciferase expression vector or purified PCR products that were amplified from the luciferase vector and co-transfected with Renilla luciferase expression vector. Empty DNA represent either the vector or the PCR product without the inserted NF-κB sequence. The cells were treated with either IL-1α or TNF-α for 4 hrs as previously described. Luciferase activity levels were quantified by a luminometer and either absolute expression levels (**C**) or fold increase over control (no treatment) are shown (**D**). Data are Mean ± SEM. *p < 0.05, *** p < 0.001.

Enhanced dynamic response to the cytokines can be improved by varying the minimal reporter region and copies of the response elements (Figure 2A). Using a forward primer of the PCR to target further minimal region of CMV promoter (-53) fused with four copies of NF-κB sites led to higher stimulation ratios (14–18 fold; ANOVA, p < 0.001) from the transfected PCR products when compared to PCR products with 2 copies of NF-κB and -95 minimal CMV promoter (Figure 2B).

In order to further evaluate the difference in response of reporter plasmids versus the reporter PCR product, both dynamic response and absolute expression levels were evaluated. The dual firefly/renilla-normalized luciferase pGL3 system was performed using the transfected PCR product or the source plasmid each harboring three copies of NFκB. Though the absolute expression levels of the reporter expressed from PCR products were lower (expectedly) than those from the plasmid, the levels were still significant and quantifiable (Figure 2C). More importantly, the dynamic and specific response of the linear reporter PCR product is superior than the plasmid. TNF-α caused enhnaced response of the NFκB in case of the NfκB-harboring PCR product when compared to mutant NFκB, in contrast to the plasmid (Figure 2C). Treatment of either IL-1α or TNF-α, caused specific and higher dynamic response (ANOVA, p < 0.001) of the luciferase reporter generated from the PCR product when compared to the plasmid (Figure 2D).

The transfection efficieny was evaluated using GFP fluorescence and cell count (on Hoechst dye background); the transfection efficiency of the PCR product (80% ± 10) was comparable to that of the plasmid transfection (85% ± 10) in HEK293 cell line-Similar results were obtained with flow cytometry. However, expression levels, as with the luciferase, were lower with the PCR product when compared to the plasmid but they are significantly quantifiable. Fore example, the fluorescence levels were 670 ± 14 million pixels per 10^4 ^cells transfected with the plasmid while in case of PCR products the fluorescence levels were in the range vicinity of 200 ± 10 million pixels per 10^4 ^in HEK293. Using a flow cytometry experiment, the mean value of fluorescence released from linear PCR product was 3741 and from the plasmid was 5666 in HEK cells.

### Promoter activity from metal- and interferon responsive PCR reporter product

**Figure 3:**
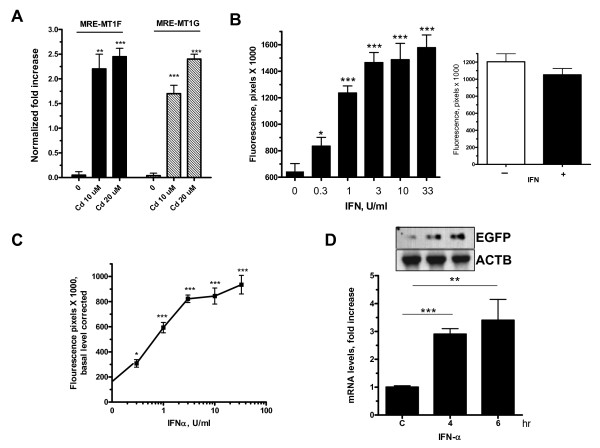
**Generation of metal and IFN-responsive reporter PCR products and their performance**. **(A) **HEK293 cells in 96-well plates were transfected with 75 ng of purified PCR products generated from reporter vector using the Forward primer that contains MRE sites (**Table 1**, *SEQ. 4 and 5*). After 20 hr, the cells were treated with 10 μM cadmium for 16 hrs. Data is Mean ± SEM (quadruplicate) – a representative experiment of two- of fold increase due to cadmium in GFP fluorescence levels that were normalized to background fluorescence from non-MRE PCR product. **(B) **Huh7 cells in 96-well plates were transfected with 75 ng of purified PCR products in which the Forward primer includes two putative ISRE sites (Table 1 *SEQ 6*). After approximately 20 hr, the cells were treated with IFN-α (log_0.5_) for an additional 16 hrs. *Right panel*: An IFN-resistant HEK293 cells were transfected with the ISRE-containing EGFP PCR product (20 hr) and then treated with IFN-α (33 IU/ml) for 16 hr. Data are from one experiment (Mean ± SEM (n = 4). **(C) **Dose-response curve for IFN action on GFP reporter activity from the ISRE-containing PCR product (normalized to fluorescence levels from non-responsive reporter). *, p < 0.01, **p < 0.005 and ***p < 0.0001. (**D**) Total RNA was extracted from Huh7 cells that were transfected with IFN-responsive reporter PCR products in the presence or absence of IFN-α (100 U/ml) for 4 or 6 hrs. RT-PCR was performed using primers specific to EGFP as described in Methods. PCR products were run on gel, and β-actin normalized signal intensities of ethidium bromide-stained products (Mean ± SEM (n = 3) were quantitated using AlphaEase.

The cloning-free approach (Figure 3A) was easily applied to generate a PCR product expressing EGFP reporter under the control of metal responsive element (MRE)-mediated transcription. Minimal metal response elements [8] that belong to metallothioneins promoters, MT1F and MT1G (Table 1), were included in the Forward primer. The heavy metal, cadmium, was used to upregulate the transcription of the MRE-containing minimal promoter from the transfected EGFP reporter PCR product (Figure 3A). At 10 uM of cadmium, response from MT1F, which contains three MRE sites was stronger than that of MT1G, which has two MRE sites. However, at 20 uM, both MREs mediate similar responses.

A minimal IFN-response element (ISRE) from the IFN-stimulated gene, ISG56 (IFIT1) [9] was incorporated in the forward primer of the PCR. IFN-α was able to induce the transcriptional activation of the transfected linear EGFP PCR product that contains the ISRE-containing minimal promoter, in the IFN-sensitive Huh7 cell line (Figure 3B). In contrast, the transcriptional activation by IFN-α from the ISRE-bearing PCR cassette was not observed in a Stat1 response-deficient cell line, 293 clone (Figure 3B, ***right panel***). As low as 0.3 U/ml was able to induce reporter activity due to transcriptional stimulation by IFN. Maximum stimulation (~5-fold) was observed at 33 U/ml of IFN-α (Figure 3C). At mRNA levels, IFN strongly induced ISRE-responsive reporter activity from the transfected PCR product at 4 hrs post-treatment (Figure 3D).

### Evaluation of minimal constitutive and inducible sequences

**Figure 4:**
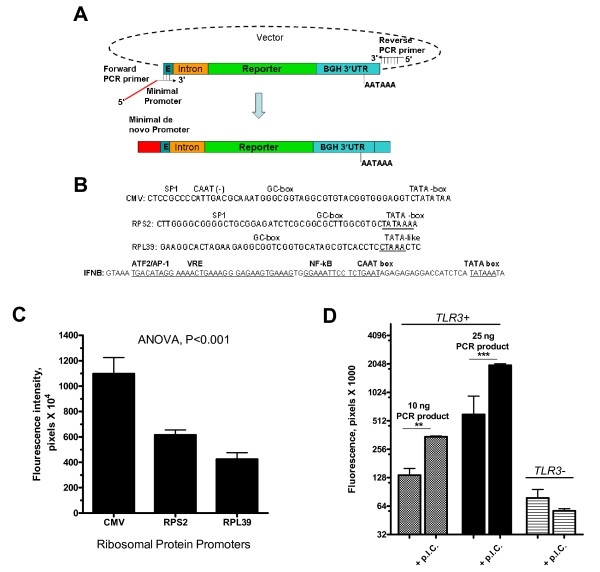
**Cloning-free construction and evaluation of de novo minimal promoters**. **(A, B) **The reporter expression plasmid was used as a template for PCR. Forward primer contains at 3' end, 18 bases that target 5'UTR (exon 1 of the CMV IE gene) upstream of reporter cDNA and 5' end sequence representing 48 bases derived from CMV (control), RPS2, RPL39 promoters or the minimal inducible IFNB promoter (**Table 1**, *SEQ 7–9*). The reverse universal primer targets a region downstream of the polyA site. **(C) **The resultant and purified PCR products were transfected onto HEK293 cells. After 48 hrs, the GFP fluorescence was quantitated as outlined in the Figure 1 legend.**(D) **TLR-3 stably transfected HEK293 cells (TRL3+) or control (TLR3 negative) HEK293 cells were transiently transfected with IFNB minimal promoter-harboring PCR products and were treated with or without 25 μg/ml of poly I. C. Fluorescence levels were quantitated after 20 hr as described above.

A variation of our approach was used to examine minimal promoter regions (Figure 4A). A minimal promoter sequence of interest can be incorporated in the forward primer, whereas the 3' end of the forward primer anneals to a region upstream of the reporter cDNA. In our case, the region is exon 1 (5'UTR) downstream of the CMV promoter. This strategy was applied by evaluating the previously uncharacterized promoters of ribosomal proteins, RPS2 and RPL39, and comparing them with the CMV minimal promoter as a control (Figure 4B). The CMV minimal promoter was the strongest, as expected. The RPL39 had weaker constitutive activity when compared to RPS2 (Figure 4C).

We also tested an inducible minimal promoter of *IFNB1*, which possesses virus response inducible element (VRE) [10], and was entirely incorporated in the Forward primer of the PCR (Figure 4B, ***lower panel***). The IFNB promoter is inducible with the double-stranded RNA, poly (I.C.), a viral intermediate mimic. The HEK293 cells, which are known to lack TLR-3, are not responsive to poly (I.C.) [11], thus, we used TLR-3 over-expressing HEK293 cell line. Here, poly (I.C.) was able to increase the minimal IFNB promoter by 5-fold in HEK293 cells that over-express TLR3 (Figure 4D). There was a basal level of IFNB promoter which is likely due to over-expression of TLR3, but was further attenuated with the use of lower input of the reporter construct (Figure 4D).

### Cloning-free conversion of genes into inducible-repressible expression cassettes

**Figure 5:**
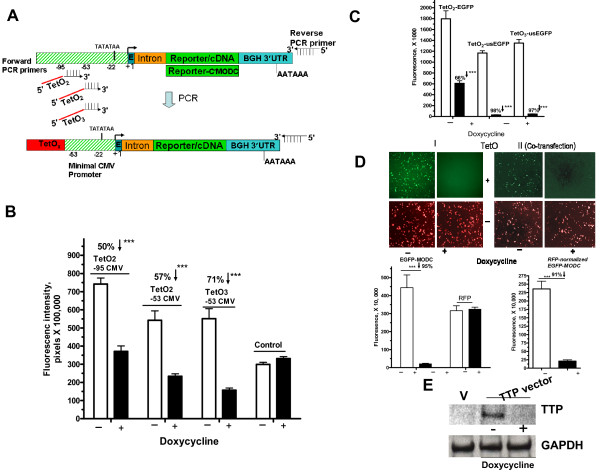
**Cloning-free conversion of genes into inducible-repressible expression cassette**. (**A**) Schematic representation of the cloning-free conversion of a gene product into tetracycline-inducible gene. Forward primer contains 3' end sequences that targets a minimal region in the vector template and 5'end that contain two or three copies of TetO sites (**Table 1**, SEQ. *10–12*). Reporter-C' MODC denotes unstable EGFP version that was created by fusing EGFP with MODC C-terminus as described in Methods. **(B) **HeLa Tet-off cells in 96-well plates were transfected with 75 ng of PCR products generated from reporter vector (**A) **and subsequently treated with or without 0.1 ug/ml of doxycycline for 26 hr. As a control, EGFP PCR product which lacks TetO was used. Arrows denotes % reduction. **(C) **Purified PCR products amplified from expression vectors that contain either wild type EGFP or unstable EGFP were transfected onto HeLa Tet-off cells in the presence or absence of 0.1 ug of doxycycline for 26 hrs. Data in B and C are Mean ± SEM (quadruplicate) from a representative experiment of three. **(D) **Representative images of the effect of doxycycline on the TetO-containing reporter expression PCR products with or without normalization of RFP-expressing PCR product that lack TetO sites. Images represent single (I) or co-transfection (II) experiments. **(E) **HeLa Tet-off cells were transfected with purified PCR products that were amplified from TTP expression vector with TetO_2 _forward primer (**Table 1**, *SEQ. 11*) and polyA reverse primer, in the absence or presence of 0.1 ug/ml of doxycycline. Western blotting using anti-body to TTP and GAPDH was performed. V denotes vector only.

The EGFP expression vector was used as a template to generate a Tet-Off/On linear cassette by the single PCR method (Figure 5A). Linear constructs were generated by PCR that contains different TetO copies and minimal promoter arrangements. The design of the forward primer, through 3' end sequence, allows flexibility in choosing the minimal sequences of the CMV promoter. The targeted regions are either -53 or -95 bases upstream of the site of transcription in the source DNA while the 5' end part allows inclusion of two or more copies of the TetO (Table 1). The PCR products are transfected into a HeLa-Tet off cell line that constitutively expresses the transactivator, tTA. In the presence of the tetracycline analog, doxycycline, tTA is inactive. In the absence of doxycycline, the tTA is active, and binds TetO containing promoter and thereby activates transcription of the linked gene. The TetO_2_-minimal promoter (-53) containing construct had slightly increased suppression in the presence of doxycycline when compared to the construct with extended (-95) minimal promoter (Figure 5B). The transfected PCR products with three copies of TetO resulted in greater repression (71%) compared to those with two TetO copies (~57%; Figure 5B). Doxycycline has no effect on EGFP linear control constructs that lack TetO sequences (Figure 4B). The moderate reduction of the EGFP is likely due to the long half life of EGFP. Subsequently, we rendered EGFP unstable by fusing with mouse ornithine decarboxylase (MODC) domain leading to shorter half life (<4 hr). There was almost complete (98%) repression (ANOVA, p < 0.001 Bonferrino post test) with the unstable EGFP when compared to the wild type GFP (66% repression) (Figure 5C). Both TetO_2 _and TeO_3 _yielded the same results (97–98% suppression, p < 0.001) in case of the unstable EGFP (Figure 5C). Doxycycline also had no effect on either stable (Figure 5B, *right columns*) or unstable EGFP that lacks TetO sites (Data not shown). Representative images of TetO-regulated experiments are shown with and without of RFP-normalized quantitation (Figure 5D) indicating that, in general, normalization is not needed as shown previously in Figure 5C. Further, we use the TeO PCR-mediated conversion approach to turn off the ectopic expression of the RNA binding protein, tristetraproline (TTP). The HeLa tet-off cells which lack any detectable TTP protein expressed TTP from the transfected PCR product that was amplified in a similar manner as the reporter. Addition of doxycycline completely turned off TTP expression (Figure 5E). Converting any reporter or gene product into a tunable TetOn-Off expression system with a single PCR is evidence of the remarkable simplicity of the described approach.

## Discussion

Monitoring gene expression in a number of applications using reporter genes is a widespread research tool. The described approach provided a reporter technology for manipulating promoter and transcriptional regulatory elements, including introduced mutations and genetic variations, without the need for the time-demanding cloning steps. The ease of making these linear reporter constructs and simplicity of introducing the desired regulatory sequences, make this reporter technology versatile and amenable to high-throughput applications. The performance of the described technology is reproducible with various combinations of promoters, reporters (here, EGFP and luciferase), cis-acting elements, minimal promoters, cell lines, and inducers. Furthermore, the described cloning-free concept was applied to a regulated gene system, specifically, the TetO inducible/repressible expression system.

The CMV minimal promoter was used in conjunction with several cis-acting elements, namely, NF-κB, ISRE, MRE, VRE, and TetO. NFκB is a member of the rel family of transcription factors that regulates several important physiological processes, including immune responses, inflammation, cell growth, apoptosis and tumorigenesis. Many different stimuli have been identified that activate the NF-κB pathway such as the pro-inflammatory cytokines TNF-α and IL-1. Both cytokines increase the reporter activity from the transfected NF-κB-regulatable PCR product generated by the described technology. Since many tumor cell lines are high in NF-κB activity [12], we have observed higher reporter activity in the cell line used in case of the NF-κB-regulated, but not the NF-κB mutant harboring linear construct.

In most of the experiments, only two copies of either NF-κB or ISRE were assessed, giving response in the vicinity of five fold. The response of the reporter assay can be improved through the use of multiple copies of the cis-acting elements [13] and use of de-stabilized forms of reporters [14,15]. Additionally, the minimal CMV promoter region can be attenuated if the target region of the forward primer is downstream towards the TATA box. A forward primer with four copies of NF-κB that also target -53 of CMV minimal promoter region results in enhanced reporter response to the cytokines (e.g., Figure 1h and 4b). This feature of the variable positioning of the target region of the forward primer allows versatile manipulation of transcriptional control elements of any promoter and production of a PCR product harboring the desired variation.

The method was applied in another cellular model, IFN response, by incorporating the ISRE of the strong IFN-responsive ISG56 gene (IFIT1)[9]. ISRE is a consensus sequence that exists in the promoter of IFN-stimulated genes and binds certain members of the IFN-regulatory factors (IRF) [16]. The IFN response of the transfected ISRE-controllable linear PCR product was observed in the IFN-sensitive Huh7 cell line but not in the 293 cell clone. This cell line clone is resistant to IFN at doses ≤ 100 U/ml [17].

Many of the genes in humans and other organisms are of unknown function, and evaluation of their promoters offers valuable information. Also, mapping of minimal promoter regions of complex gene structures and rearrangements requires a versatile approach. The ability to evaluate any sequence of interest for the ability to elicit the reporter transcription using the described PCR strategy is of notable application. We have used this approach to evaluate the differential ability of the minimal promoter sequence of RPS2 and RPL39 to induce the reporter activity. The RPL39 minimal promoter which lacks both the typical TATAAA signal and SP1 site when compared to RPS2, although it possesses TAAA, has weaker constitutive reporter activity when compared to RPS2. An entire minimal IFNB inducible sequence [10] was incorporated into the forward primer of the PCR and was responsive in TLR3-expressing 293 cell line but not in 293 cells that do not express the TLR3 gene and thus is not responsive to the viral intermediate mimic, poly (I.C.) [11].

Although any reporter can be employed with this approach, we mainly used the EGFP system. We also used, but to a lesser extent, the luciferase system. Particularly when coupled with advanced imaging processing, the EGFP is sensitive and has a large dynamic range. Use of fluorescent reporters have several advantages including repeated measurements in live cells, single cell assessment, reduced variability and ease due to the absence of cell lysis. The EGFP reporter assay resulted in intra-well variance in fluorescence that was mainly <6%, which does not warrant intra-well normalization of transfection [18], although some of the experiments utilized red fluorescent protein (RFP) for the purpose of confirming this normalization feature. There are many recent improvements in fluorescent proteins and their biophysical characteristics that render them more sensitive (brighter) and with different colors [19] that can also be used with the described approach.

The most important feature of the described method here is the enhanced applicability since it involves no cloning. An additional advantage of the use of PCR products in contrast to plasmid in transfection is the absence of plasmid preparation. Although, transfection with PCR products is generally less efficient than with plasmids, successful results are obtained with optimized expression cassettes [18,20], particularly when coupled with a sensitive reporter system and use of advanced image segmentation methods as in the case of fluorescent proteins. Other added advantages are lower cellular toxicity and extended expression [18,20,21].

Further advantages of the reporter PCR method is attributed to lack of certain characteristics that commonly encountered with plasmids use in gene and reporter expression studies, namely, cryptic transcription and supercoiling-quality variations. The elimination of the plasmid backbone may results in reduction of cryptic transcriptionally active sequences that normally found in the plasmid backbone [22] leading to more specific and larger dynamic response. Supercoiling in general is known to have positive effect on transcription, and supercoiled plasmids may be more efficient for transient mammalian gene expression when compared to linear DNA; however, there are reports that show that this is promoter-specific, and supercoiling effect was not seen with certain promoters [23,24]. Thus, the use of the PCR products at least minimizes the variations of supercoiling quality of plasmids.

A limitation is that only short transcriptional regions (<150 bases) of a large promoter can be studied. However, most of the regulatory cis-acting elements are typically short of 5–15 bases [5] and transcriptional studies rely on smaller regions that can be accommodated with the targeting position and length of the oligonucleotides. Moreover, additional PCR reactions can append additional promoter regions as required. Studies with deletion promoter mutants can also benefit from the cloning-free strategy by designing primers that target sequential regions of the promoter.

Converting any gene product from an expression vector into an inducible/repressible expression linear construct is made possible by the cloning-free linear DNA construction. In this study, we utilized the common TetO system in which the tetracycline-responsive TetO elements are regulated by the repressor, rtTA, or the activator protein, tTA the Tet-On and Tet-Off systems [2]. Significant leakage reduction and gene repression was achieved by the TetO-PCR product transfection, particularly with the use of unstable EGFP and three copies of TetO leading to total repression.

## Conclusion

This study reports several cloning-free strategies to generate transcriptionally-regulatable PCR products that when transfected express measurable reporter activity. There are many other variations and subsequently versatile applications for the described approach. Tunable manipulations in gene and reporter expression such as the number of transcriptional element copies, mutations, gene destabilization elements, and minimal region are made straightforward by the described approach. The versatility and ease of making the linear reporter constructs for transcriptional studies should make this approach widely used and suitable for high throughput applications. This cloning-free system is utilized to fine tune the transcriptional control of any gene of interest and it makes transcriptional assessment studies and converting genes into inducible/repressible systems a simple task.

## Methods

### Cell lines and inducers

HEK293 was obtained from American Type Culture Collection (ATCC; Rockville, MD) and cultured in RPMI 1640 (Invitrogen, Carlsbad, CA) supplemented with 10% FBS and antibiotics. Huh-7 cells were obtained from Dr. Stephen Polyak (University of Washington, Seattle, WA) and propagated in DMEM medium with 10% FBS and antibiotics. The TLR-3 over-expressing HEK293 cell line was purchased from Cayla-Invivogen (France). HeLa-Tet off cell line which stably expresses tTA was obtained from Clontech, Inc. (Mountain View, CA) and was grown in DMEM medium using Tet-approved serum (Clontech) and maintained with the selection drug, G418. Recombinant human IL-1α and TNF-α were obtained from R & D systems (Minneapolis, MN) and Human rIFN-a2a (Roferon) was from Hoffman-LaRoche, Basel, Switzerland and had specific activity of 2 × 10^8 ^IU/mg. Cadmium sulfate and poly(I.C.) were purchased from Sigma (St Louis, MO). Doxycycline was obtained from Sigma.

### Plasmids, plasmid constructions

The EGFP-Gwiz plasmid which is under the control of CMV/Intron A was obtained from Genelantis (San Diego, CA). The same plasmid backbone was used to construct other forms of reporters. The wild type EGFP was rendered unstable by fusing with the amino acids 422–461 of the degradation domain of mouse ornithine decarboxylase (MODC). Briefly, the MODC domain was amplified from genomic DNA of mouse fibroblasts using specific primers that contain EcoRI and BamH1 sites in the forward and reverse primer, respectively. The amplified cDNA was cloned in frame with EGFP coding region using the same restriction sites. The RPF-Gwiz plasmid was constructed by first amplifying RFP cDNA from TurboRFP (Evrogen, Russia) with primers containing SalI and BamHI sites in the forward and reverse primer, respectively. Subsequently, the amplified RFP cDNA was cloned into Gwiz plasmid that was previously cut with SalI and BamH1 sites. Constructs were verified by both sequencing and expression. TTP cDNA was excised from bluescript vector (obtained from Dr. Perry Blackshear, NIH) and cloned into a pcDNA3.1 vector. The pGL3-NfκB was obtained from Dr. M. Dehbi (King Faisal Specialist Hospital and Research Center), it contains 3 copies of NfκB cloned into pGL3 using NheI-XhoI sites upstream of minimal SV40 promoter.

### Expression Active reporter PCR

The expression active PCR products were generated directly from the vector using two primers, a forward primer that targets vector sequences (See Oligonucleotides below) and a reverse primer that is complementary to a downstream region of the poly(A) site. The latter is as follows: 5' CCA TAG AGC CCA CCG CAT 3'. The PCR is generated from template that is not complex, i.e., plasmid DNA, the PCR -also with annealing temperature of 51°C. Thus it is straightforward and robust with the forward primer that has 17–19 bases of complementary sequence to the plasmid template. PCRs were carried out using the following reagents and conditions: 2.5 U HotStart Taq (Qiagen) and 0.2 U Pfx polymerase (Invitrogen, Carlsbad, CA) mix, 2 μl (100–200 ng) of the vector template, 1× PCR buffer, 0.2 mM dNTPs, 0.2 μM primers, with the following cycle conditions: 95°C for 12 min, 31 cycles of: 94°C, 1 min., 51°C, 1 min., 72°C, 4 min., and final extension at 72°C for 7 min. The PCR products were purified using Qiagen PCR purification columns to eliminate the primers, small PCR products, buffer, and enzymes. The PCR products were finally eluted in sterile water. The typical yield of purification is 0.2 ug/ul in a volume of 30 ul. The PCR products were run on a 1.2% agarose gel and visualized by ethidium bromide under UV light to verify size and quality.

### Oligonucleotides (Forward Primers)

The forward primer used for reporter expression active PCR contains 3' end sequences (~18 bases) complementary to a desired region in the vector template upstream of the EGFP coding region and 5' end sequences that accommodate any further described transcriptional control elements (Table 1). HPLC-purified oligonucleotides were custom-synthesized by Metabion (Germany).

### Transfection experiments

Cells were grown at standard culture conditions (37°C, 5% CO_2_). 3 × 10^4 ^cells per well in 96-well clear-bottom black plates (Matrix technologies, Hudson, NH) were transfected with purified reporter linear constructs (PCR products). Transfections were performed in serum-free medium using Lipofectinamine 2000 (Invitrogen). All transfections were performed in several replicates as indicated in the text. The variance in GFP fluorescence among replicate microwells was < 6%; thus, with this minimum variance, experiments do not warrant transfection normalization [18]. Data are presented as mean values ± standard error (SEM). Western blotting of the transfected cell line was used in the case of the RNA binding protein, TTP, and GAPDH. Rabbit anti-TTP antibody was obtained from Santa Cruz, CA, and used at 1/500. Secondary HRP-conjugated antibody (1/1000) was used.

### Luciferase assay

Cells were grown at 3 × 10^4 ^cells per well in 96-well plates and were transfected with purified reporter linear constructs (PCR products). After treatment, cells were harvested by aspiration of the media followed by the addition of lysis buffer (Promega) per well and transferred to an opaque 96-well plate. Luciferase assays were performed using the Dual-Luciferase Reporter Assay System (Promega) according to the manufacturer's instructions and measured on a ZENYTH 3100 luminometer. Data are presented as fold increase over basal levels according to the readings of Mean ± SEM of luminescence.

### Semi-Quantitative PCR

Total RNA was extracted using Trizol method. RT reaction was performed using 200 ng total RNA, 500 ng oligo dT_(18–23)_, 500 mM dNTP mixture, 20 U RNAsin (Pharmacia), 200 U of SuperScript II (Invitrogen). Hot start PCR amplification was performed using HotStart Taq DNA polymerase (Qiagen). The cDNA was amplified at optimum cycle number according to an amplification curve of EGFP and β-actin cDNA that was determined for each cDNA of interest by plotting increasing cycle numbers against ethidium bromide stained gel intensity of the amplified products. Cycling was 94°C for 60 s, 60°C for 30 s, and 72°C for 60 s using primers specific to EGFP mRNA and β-actin mRNA. The EGFP primers are forward: 5' CCACGCTGTTTTGACC TCCA3' and reverse primer: 5' CACCCTCTCCACTGACAGAGA 3' which give PCR product of 230 bases. The β-actin forward primer is 5' ATCTGGCACCACACCTTCTACAATGAGCTGC G3' and the β-actin reverse primer: 5' CGTCATACTCCTGCT TGCTGATCCACA3'. Both primer pairs were designed to span intronic sequences so that the larger PCR products due to genomic DNA contamination are not favored in amplification. Quantitation of ethidium bromide-stained signals on agarose gels were performed by AlphaEase software (Alpha Innotech, San Diego, CA).

### Imaging fluorescence measurement and quantitation

Efficiency and level of transfection was aided by monitoring the fluorescence from EGFP constructs (optimum excitation wavelength: 488 nm and emission wavelength: 503 nm). Pictures were taken automatically using BD high-throughput imaging system, BD Pathway 435 (BD Biosciences, Europe. In all cases, exposure intensity and duration, PMT gain, and other settings were kept constant to allow equal comparison of experiments. Auto-focus of the instrument was performed for each image in 2 × 2 montage. Automated identification of cell regions was performed using improved in-house software, ProXcell. The green o red bands of the captured color images of cells consisting of ~1344 × 1024 pixels were processed as follows. The images were divided into four quadrants and each quadrant was processed and analyzed separately to speed up the process. Morphological opening process was utilized to obtain the non-uniform background illumination pattern. Further, images were filtered using an anisotropic diffusion filtering technique [25] which is a non-uniform filtering approach that preserves the image sharpness and removes the noise. The cells were segmented from the background using a Markov Random Field based segmentation technique [26] The total cell and well area (pixels), total green intensity within the cell regions were calculated and saved into an ASCII file in tab-delimited format for further processing. The graphical user interface (GUI) that integrates the whole process was implemented in MATLAB (The Mathworks, Inc., Natick, MA, USA) programming environment, and the 96-images were processed in batch mode.

Data are presented as Mean ± SEM of total fluorescence intensity in each well with replicate readings ranging from three to eight as indicated in the text. Student t'test was used when comparing two data groups while analysis of variance (ANOVA) was performed for each data set having three or more data groups.

## Authors' contributions

LA performed experiments and analyzed the data. WA performed experiments and analyzed the data. MA carried out cloning and performed some of the experiments. OD developed the imaging segmentation and analysis algorithm. KSA conceived and designed the study, and wrote the paper. All authors read and approved the final version of the manuscript.
